# Identification of focal ARDS using ventilatory ratio

**DOI:** 10.1186/s13054-021-03796-y

**Published:** 2021-10-24

**Authors:** Kay Choong See, Melanie Torres Estaras, Juvel Mabao Taculod

**Affiliations:** 1 Division of Respiratory and Critical Care Medicine, Department of Medicine, National University Hospital, 1E Kent Ridge Road, NUHS Tower Block Level 10, Singapore, 119228 Singapore; 2 Department of Medicine, Yong Loo Lin School of Medicine, National University of Singapore, Singapore, Singapore; 3 Division of Critical Care – Respiratory Therapy, National University Hospital, Singapore, Singapore

**Keywords:** Diagnosis, Lung, Respiratory distress syndrome, Adult, Ultrasonography

**Dear Editor**

Acute respiratory distress syndrome (ARDS) patients with disease predominantly in the posterobasal lung regions (i.e., focal ARDS) benefited from prone positioning, while patients with diffuse (non-focal) ARDS benefited from recruitment maneuvers and high positive end-expiratory pressures, provided focal ARDS was correctly classified [1]. Classification of ARDS morphology using imaging is however challenging, as computed tomography is resource intensive, and lung ultrasound is operator dependent.

Alternative methods for focal ARDS identification are therefore needed. Our prior study using partial pressure of arterial oxygen divided by fraction of inspired oxygen (P/F ratio) did *not* allow the identification of focal ARDS morphology [2], suggesting that the degree of oxygenation impairment is related to the *extent* rather than the *distribution* of lung involvement. Another physiological parameter—the ventilatory ratio (VR), as an estimate of dead space fraction [3]—holds promise. Compared to patients with diffuse ARDS, patients with focal ARDS had lower physiological dead space, which was computed according to the Enghoff modification of Bohr’s equation [4]. We therefore hypothesized that VR could help to identify focal ARDS.

Patients were included if they had ARDS fulfilling the Berlin Definition and received invasive mechanical ventilation. On admission, trained respiratory therapists performed 12-point lung ultrasound using a 2–4 MHz phased array transducer and semi-quantitatively scored each region [5]. We identified focal ARDS on lung ultrasound, if the consolidated regions were only present in the posterobasal regions and absent in the anteroapical regions [1, 2]. VR, a dimensionless variable, was computed as (minute ventilation × partial pressure of arterial carbon dioxide)/(predicted body weight × 3750) [3].

The association of focal ARDS with VR was analyzed assuming a nonlinear relationship. A logistic regression model was fitted using a restricted cubic spline with four knots and taking the VR of the first knot as the reference level. Should the spline suggest a VR threshold for prediction of focal ARDS, we proceeded to elucidate this threshold by performing binary logistic regression using focal ARDS as the independent variable and VR threshold as the dependent variable, with the latter tested in 0.1 intervals.

A total of 152 patients were studied (age 63.3 ± 14.1 years; 53 (34.9%) female; mean P/F ratio 148 ± 71 mmHg; mean VR 2.18 ± 1.19; ICU mortality 16.5%; hospital mortality 33.6%). Sixteen (10.3%) had focal ARDS. Admission diagnoses were as follows: pneumonia (61 patients; 40.1%), non-pneumonia sepsis (19; 12.5%), chronic obstructive pulmonary disease (9; 5.9%), acute myocardial infarction (3; 2.0%), stroke (12; 7.9%), other diagnoses such as massive hemoptysis, pulmonary vasculitis and pneumonitis (48; 32.6%). Median lung ultrasound scores (interquartile range) were as follows: right posterobasal 3 (0–6), left posterobasal 2.5 (0–5), right anteroapical 0 (0–3), left anteroapical 2 (0–3).

Spline analysis suggested a threshold effect (Fig. 1). A VR of < 1.2 was associated with focal ARDS (odds ratio 3.41, 95% confidence interval 1.05–11.1, *P* = 0.041), with sensitivity 31.3%, specificity 88.3%, positive predictive value 23.8%, and negative predictive value 91.6%. As such, VR > 1.2 could help exclude focal ARDS and aid personalized ARDS management, though our preliminary findings require external validation.

**Fig. 1:**
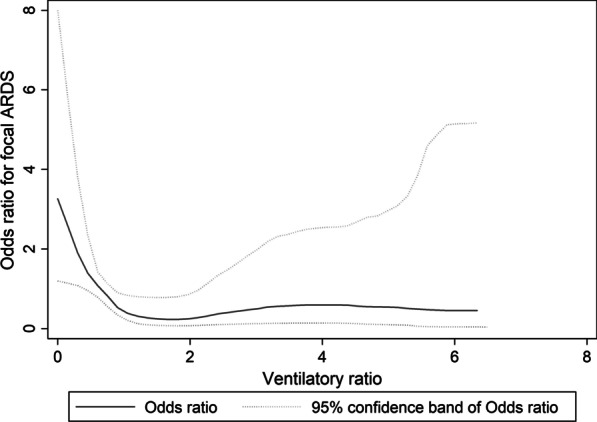
Association of odds ratio for focal ARDS with ventilatory ratio, using a restricted cubic spline with four knots

## Data Availability

The data that support the findings of this study are available from the corresponding author, KCS, upon reasonable request.

## Abbreviations

ARDS: Acute respiratory distress syndrome
P/F ratio: Ratio of partial pressure of arterial oxygen divided by fraction of inspired oxygen
VR: Ventilatory ratio
